# Mothers, Babies, and the Little Bill

**Published:** 1918-09-21

**Authors:** 


### THE WOMAN WORKER'S WORLD.

### Mothers, Babies, and the Little Bill.

MR. HAYES FISHER'S circular* to Councils other than the L.C.C. and Sanitary Authorities, of which we published a summary in our issue of August 31, should be read by others besides officials. Even those who are most disappointed that a very little Bill comes in place of the overdue Ministry of Health must recognise that if the permissive powers given are really used much can be done towards the creative and co-ordinating task for which that Ministry is needed. Grant, for instance, is payable to voluntary societies on condition that their work is linked with that of the local authority, and voluntary schemes are preferably to be submitted through the authority itself. The weakness of the permissive condition lies in the fact that there are slothful Councils which will not act unless pressure is brought to bear. The fact that two at least of the members of committees appointed to work the Act must be women should prove a stimulus to organisations of women, Institutes, Co-operative Guilds, Women Citizen Councils, etc., to move in the matter. The President of the Local Government Board emphasises the need for working women on such committees, and recommends application for candidates to such local bodies of women or to the Standing Committee of Industrial Women's Organisations (33 Eccleston Square, London).

### PROBLEMS IN MIDWIFERY SERVICE.

Let it be said at once that the pressing question of the practising midwife's livelihood is not set at rest, though some contribution towards it is made by the grant available for salary and expenses of inspectors of midwives, who should be medical women, or highly trained midwives who can instruct others, and to institutions and charities running a midwifery service at cheap rates for deficits in their expenses, also for "the provision of a midwife for necessitous women in confinement and for areas which are insufficiently supplied with this service." (Payments to the C.M.B. and compensation to midwives thrown out of work by infection are categorically excluded.) On the interpretation of this regulation much difficulty must arise. What is a necessitous woman? What constitutes insufficient supply? If in an area of long distances, can the service be extended by the use of that risky aid the motor-cycle? If in a crowded slum where the black flag has been run up, or one or two practitioners are laid low by Spanish influenza, will the temporary deficiency be met? The best brains, plus a close knowledge of local conditions, will be needed for the important unpaid service to be rendered by members of administering committees.

### THE TRAINING OF HOME HELPS.

A forward step is taken by grant being payable alike towards increased accommodation in lying-in homes and for the training in cookery, child-care, mending, etc., at Maternity and Child-welfare Centres, for a term of one to three months, of women as home helps in confinements taking place in the mothers' own rooms. Where necessary, the children may even be boarded out. Such cases involve specially careful visiting, and very careful selection as well as training of the helps, as well as tactful regard for the mother's own wishes. We have heard of a case in which a pair of fine violet eyes counted as a serious objection! But a woman gifted with knowledge and tact might do wonders by simply showing how existing difficulties could be conquered.

A new hope for the sorely handicapped, unfathered babe dawns with the gift of help to keep mother and child together or to complete payments to a *good* foster-home, the cheap one involves either exploitation of the infant or at least squalor. Here, again, is a great chance for elevating effort on the part of a visitor, and we may also hope that experience will point the way to better official and legislative dealing with the fathers.

### HEALTH VISITORS' TRAINING STILL UNSTANDARDISED.

Though the functions of the Health Visitor are clearly defined, and a reduction of her work from an average of 500 to one of 400 births is desired, the standardisation of her training is not attempted. It is, however, stated that qualifications ranging from a medical degree to the previous discharge of similar duties under a local authority will be needed to secure the grant towards salary, unless the Board have other "adequate evidence" of fitness. A voluntary worker recommended by the medical officer may, however, obtain a grant for training.

### ENCOURAGING POSSIBILITIES.

Dental attention and ophthalmic work for mothers and under-fives at Centres may obtain grant, and the cost of dentures or spectacles may, if necessary, be wholly or partly remitted. While the permissive character of such an Act makes it possible to drive—a perambulator through its provisions, great possibilities are opened by the grants available for treatment and teaching at Centres, for hospital treatment of young children, for food, for care of under-five children of widows, of deserted and unmarried mothers, convalescent homes, experimental work, and contributions to voluntary agencies and institutions.

* *Maternity and Child Welfare* (4). H.M. Stationery Office, Imperial House, Kingsway, W.C. Price 1d.

### £15,000 for Welfare Institutions.

The work of the National League for Health, Maternity and Child Welfare, 4 Tavistock Square, secretary Miss J. Halford, will consist largely in allocating the £15,000 grant of the American Red Cross, for the formation and maintenance for one year of sixty Infant Welfare institutions. Two-thirds of this sum is to be spent on maternity hostels, providing one each for England, Scotland, Ireland, and Wales, as well as London. The rest of the money will be largely devoted to the maintenance of ante-natal clinics, of which the League will hope to have at least ten in London and twenty in the provinces, paid for by it and established in connection with existing Infant Welfare Centres. Day nurseries in factories as organised in France will also be tried. Money will also be spent on an emergency home for babies whose mothers are temporarily disabled by illness, or waiting a vacancy elsewhere, and on a day nursery for children of the professional classes whose mothers are working to supplement separation allowance. The League now includes the National League for Physical Education and Improvement, the National Association for the Prevention of Infant Mortality, the Association of Infant Welfare and Maternity Centres, with the coming addition of the National Baby Week Council, National Society of Day Nurseries, and Women's Imperial Health Association.

